# Decreased expression of prolyl hydroxylase 1 is associated with poor prognosis in colorectal cancers

**DOI:** 10.1007/s00432-023-04717-y

**Published:** 2023-03-28

**Authors:** Nathaniel Melling, Julia Grass, Matthias Reeh, Michael Tachezy, Marco Blessmann, Jakob R. Izbicki, Katharina Grupp

**Affiliations:** 1 Department of General, Visceral and Thoracic Surgery, University Medical Center Hamburg-Eppendorf, Hamburg, Germany; 2 Department of Plastic, Reconstructive and Aesthetic Surgery, University Medical Center Hamburg-Eppendorf, Hamburg, Germany

**Keywords:** PHD1, Colorectal cancer, Tissue microarray, IHC

## Abstract

**Background:**

Prolyl hydroxylase 1 (PHD1) is a prognostic marker in several cancers.

**Aims and scopes:**

This study was undertaken to elucidate the clinical relevance of PHD1 in colorectal cancer (CRC) prognosis.

**Materials and methods:**

We compared PHD1 expression on a tissue microarray (TMA) containing samples from 1800 CRCs with corresponding clinicopathological tumor variables and patient survival.

**Results:**

While PHD1 staining was always high in benign colorectal epithelium, high PHD1 staining was detectable in only 71.8% of CRCs. Low PHD1 staining was associated with advanced tumor stage (*p* = 0.0101) and shortened overall survival in CRC patients (*p* = 0.0011). In a multivariable analysis including tumor stage, histological type and PHD1 staining revealed tumor stage and histological type (*p* < 0.0001 each), but also PHD1 staining (*p* = 0.0202) to be independent prognostic markers for CRC.

**Conclusions:**

In our cohort, loss of PHD1 expression independently identified a subset of CRC patients with poor overall survival and might, thus, be a promising prognostic marker. PHD1 targeting may even allow for specific therapeutic approaches for these patients.

## Introduction

Colorectal cancer (CRC) is the fourth most common malignant disease worldwide (Jemal et al. 2011). Despite recent advances in the management of this disease, CRC remains the second leading cancer-related cause of death in western countries (Jemal et al. 2011).

Hypoxia is a non-physiological level of oxygen tension, that is common in most of malignant tumors (Harris 2002). Hypoxic stress leads to selective pressure in the microenvironment of tumors, and control of this restrictive circumstance is essential for tumor progression (Harris 2002). In this context, hypoxia inducible factors (HIFs) and their regulators, the prolyl hydroxylases (PHDs), play a pivotal role in cell response to lack of oxygen (Wang and Semenza 1995; Bruick and McKnight 2001). Under physiological normoxic conditions, PHDs hydroxylate the alpha subunit of HIF1 (HIF1α) at proline residues, which results in its degradation (Ivan et al. 2001; Jaakkola et al. 2001). Under hypoxia, the activity of PHDs reduced, and thus, HIF1α is stabilized, leading to robust expression of hypoxia-regulated genes (Wang and Semenza 1995). Besides its function as inhibitor of HIF1α stability, PHD1 has either tumor promoting or suppressing activity depending on the cell and cancer type-specific signaling pathways (Jokilehto and Jaakkola 2010; Seth et al. 2002; Erez et al. 2003). For example, one in vitro study demonstrated that PHD1 stimulates cell proliferation of breast cancer cells (Seth et al. 2002), while another study showed that ectopic expression of mPHD1 suppressed tumor growth in a mouse model with colon carcinoma cells (Erez et al. 2003).

Immunohistochemical studies described aberrant PHD1 expression in diverse malignancies (Couvelard et al. 2008; Gossage et al. 2010; Andersen et al. 2011; Kaufmann et al. 2013; Bur et al. 2018) and suggested a prognostic relevance of PHD1 expression in non-small cell type lung cancers (Andersen et al. 2011), pancreatic endocrine tumors (Couvelard et al. 2008), and classical Hodgkin's lymphoma (Bur et al. 2018). For colorectal cancer, immunohistochemical studies demonstrated that PHD1 expression is increased in malignant as compared to benign colorectal epithelium (Rawluszko et al. 2013; Xie et al. 2012). Functional studies have revealed that ectopic expression of PHD1 suppresses accumulation of HIF1α and that carcinoma cells expressing PHD1, which were injected into mice, inhibited tumor growth though increased necrosis and decreased microvessel density (Erez et al. 2003). Additionally, PHD1 knockdown is described to sensitize CRC to 5-FU in mice, and thus, the authors proposed that PHD1 is part of the resistance machinery in CRC (Deschoemaeker et al. 2015). To further elucidate the importance of PHD1 as a clinically relevant prognostic biomarker in CRCs, a TMA containing 1800 CRC specimens with corresponding follow-up data was analyzed for PHD1 expression.

Here, we data demonstrate that lack of PHD1 expression predicts poor prognosis in CRCs.

## Materials and methods

### Patients

**Fig. 1:**
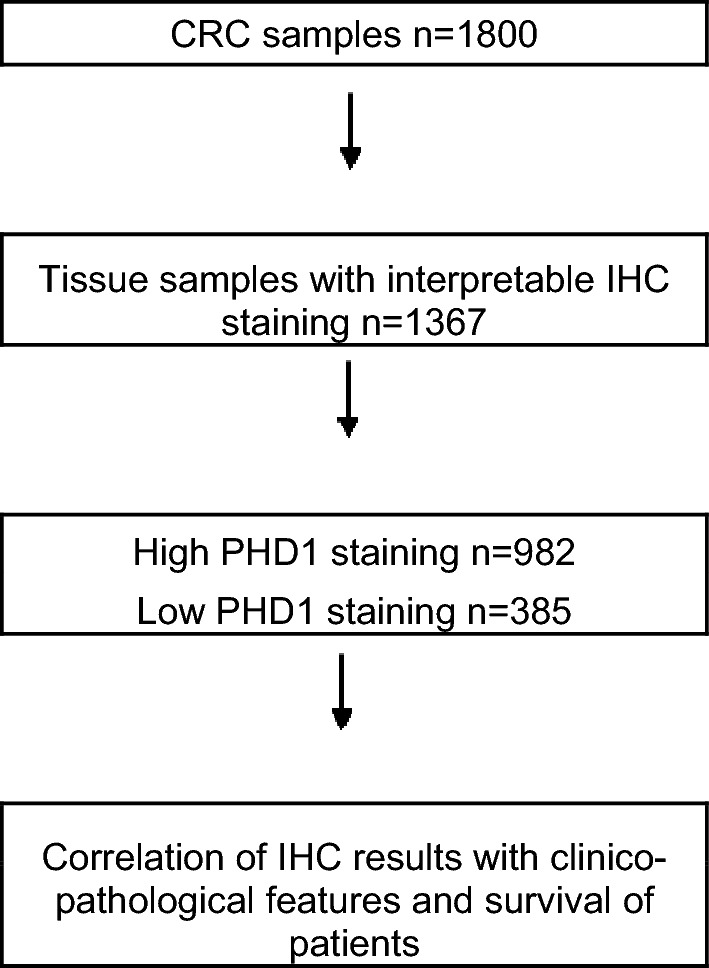
Flow diagram for the study cohort

Two TMAs with a total of 1800 CRC samples were included in this study. The first TMA was manufactured from surgical specimens of 1420 CRC patients at the Institute of Pathology of the University Hospital of Basel, while the second included 380 samples from our institution. Figure 1 shows the flow diagram for the study cohort.

All specimens were collected from patients who underwent primary surgery for colorectal cancer without neoadjuvant therapy, so as to evaluate the natural course of the disease. Postoperative therapy was executed according to the guidelines for the treatment of colorectal carcinoma (Schmiegel et al. 2010). For both cohorts, survival data and pathological parameters were complete. No data were available regarding disease progression as assessed by clinical or RECIST guidelines.

TMA construction was as described (Kononen et al. 1998). In brief, hematoxylin and eosin-stained sections were made from each block to define representative tumor regions. Tissue cylinders with a diameter of 0.6 mm were then punched from tumor areas of each “donor” tissue block using a home-made semi-automated precision instrument and brought into empty recipient paraffin blocks. Four μm sections of the resulting TMA blocks were transferred to an adhesive coated slide system (Instrumedics Inc., Hackensack, New Jersey). Patient information and clinical data, such as age, sex, localization and type of tumor, pTNM-stage, and carcinoma grade, were retrospectively retrieved from clinical and pathological databases (Table 1).

**Table 1:** Clinicopathological features of CRCs

Clinicopathological features	Available, *n*
Gender	
Male	690
Female	675
Age	Mean: 69 (29–96)
Tumor stage	
pT1	62
pT2	219
pT3	869
pT4	200
Nodal status	
pN0	725
pN1	330
pN2/3	280
Grading	
G1	20
G2	1141
G3	185
Tumor localization	
Right-sided colon	270
Transverse colon	86
Left-sided colon	315
Rectum	335
Histological type	
Non-mucinous carcinoma	918
Mucinous carcinoma	84
Others	14

In detail, 690 of the patients were male and 675 were female. The distribution of cancers according to tumor size was as follows: 62 pT1 cancers, 219 pT2 cancers, 869 pT3, and 200 pT4 cancers. 725 of the tumors were staged as nodal negative, while 610 specimens revealed nodal involvement. Regarding the tumor grading, 20 tumor samples were classified as G1, 1141 G2, and 185 G3. Patients presenting with distant metastases (UICC stage 4) were not included in our cohort. All tumors were assessed by two experienced colorectal examiners (NM, KG). Follow-up data were obtained from local cancer register boards or via attending physicians. The median follow-up time was 46 months (range 1–152 months) for the first and 36 months (range 1–179 months) for the second cohort.

For statistical analyses, tumor localization was grouped as follows: right-sided cancer (cecum, ascending colon), cancer of the transverse colon including both flexures, cancer of the left-sided colon (descending colon, sigmoid colon), and rectum. The utilization of tissues and clinical data was according to the Hamburger Krankenhaus Gesetz (§12 HmbKHG) and approved by our local Ethical Committee.

### Immunohistochemistry

Freshly cut TMA sections were analyzed during 1 day in one single analysis. Slides were deparaffinized and exposed to heat-induced antigen retrieval for 5 min in an autoclave at 121 °C in pH 7.8 Tris–EDTA-Citrate buffer prior to incubation with antibody PHD1 (polyclonal; rabbit; Novus Biologicals; 1/450 dilution). Bound antibody was visualized using the EnVision Kit (Dako). PHD1 immunostaining was analyzed by one person experienced in IHC analysis (KG). PHD1 staining was predominantly localized in the cytoplasm of the cells and was rarely accompanied by lower expression levels in the nucleus of the cells. PHD1 staining was homogenous in the analyzed tissue samples and staining intensity was semiquantitatively recorded. TMA spots revealing homogenously weak staining intensity were scored as low and spots with homogenously strong staining intensity were scored as high.

### Statistics

Statistical calculations were performed with JPM 10 software (SAS Institute Inc., NC, USA). Contingency tables and the *χ*^2^ test were performed to search for associations between PHD1 expression and tumor phenotype. Survival curves were calculated according to Kaplan–Meier. The log-rank test was applied to detect significant differences between groups, and COX proportional hazards regression analysis was performed to test the statistical independence and significance between pathological, molecular, and clinical variables.

## Results

### IHC analysis

**Fig. 2:**
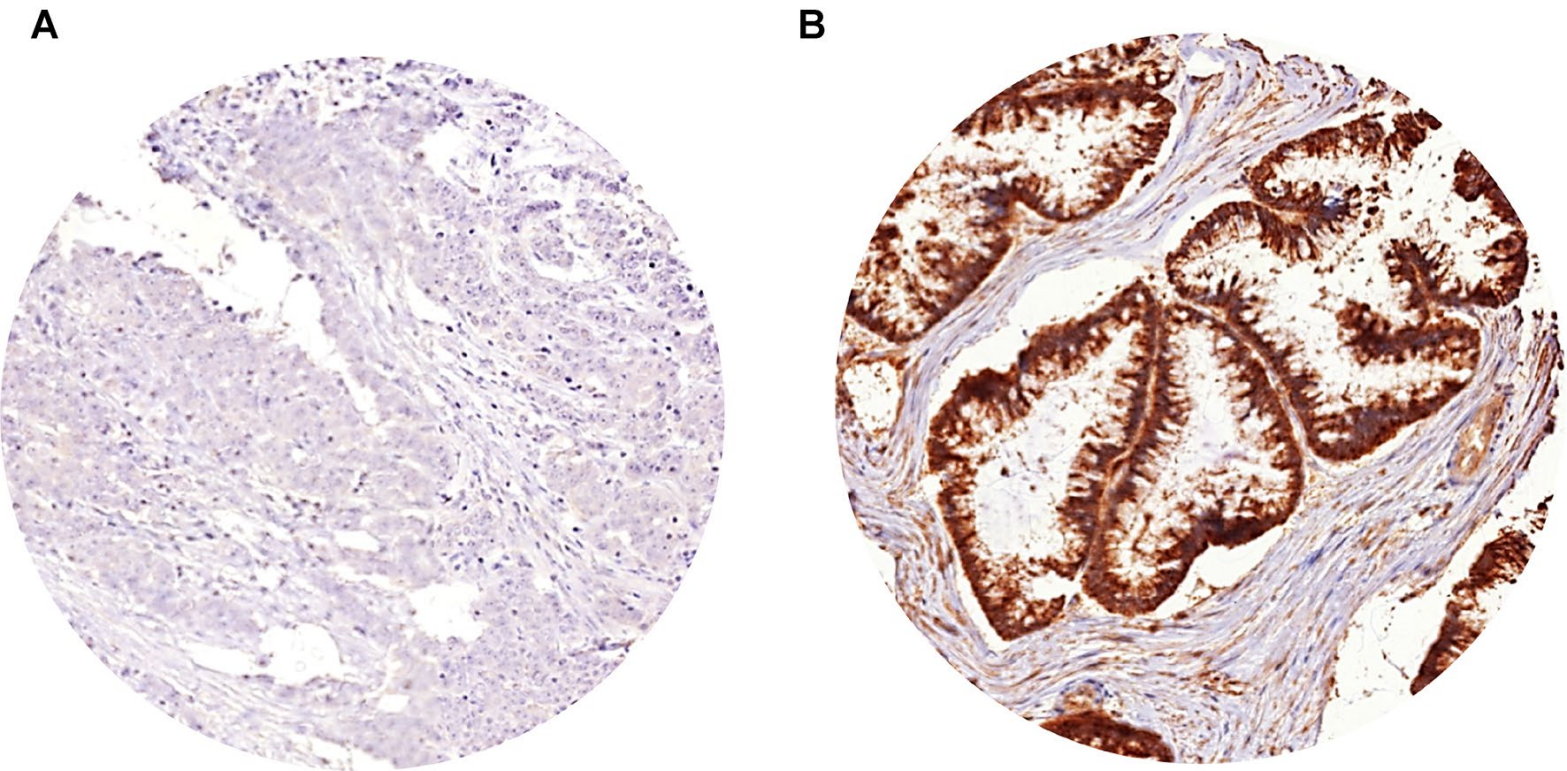
PHD1 expression in CRCs. Representative PHD1 immunostaining of **A** low and **B** high immunoreactivity in CRCs

A total of 433 of 1800 (24%) tissue spots were non-informative for PHD1 immunohistochemistry due to the complete lack of tissue or absence of unequivocal cancer cells on the respective TMA spots. All the remaining tissue samples were used for IHC analysis. Representative images of PHD1 immunostaining in CRCs are given in Fig. 2.

### PHD1 immunohistochemistry in primary CRCs

PHD1 immunostaining was predominantly localized in the cytoplasm of the cells. Cancer cells showed lower intensities of PHD1 staining than normal colorectal epithelium. In CRCs, PHD1 immunostaining was considered high in 71.8% and low in 28.2% of 1367 interpretable cases.

### Association of PHD1 expression with tumor phenotype

Low PHD1 staining was significantly associated with advanced tumor stage (*p* = 0.0101) and non-mucinous histological type (*p* < 0.0001), as shown in Table 2. However, PHD1 immunostaining was not significantly linked to nodal status (*p* = 0.2847), tumor grading (*p* = 0.1143), or tumor localization (*p* = 0.2653).

**Table 2:** PHD1 immunostaining in association with clinical parameters in CRCs

Parameters	PHD1 IHC			
	Analyzable, *n*	Low expression, %	High expression, %	*p* Value
All cancers	1367	28.2	71.8	
Tumor stage				
pT1	62	11.3	88.7	0.0101
pT2	219	27.4	72.6	
pT3	869	29.6	70.4	
pT4	200	28.5	71.5	
Nodal status				
pN0	725	27	73	0.1847
pN1	330	28.5	71.5	
pN2	279	31.9	68.1	
Grading				
G1	20	15	85	0.1143
G2	1141	29.2	70.8	
G3	185	23.8	76.2	
Tumor localization				
Right-sided colon	270	22.2	77.8	0.2653
Transverse colon	86	30.2	69.8	
Left-sided colon	315	28.3	71.7	
Rectum	335	24.5	75.5	
Histological type				
Non-mucinous carcinoma	918	27.1	72.9	< 0.0001
Mucinous carcinoma	84	5.9	94.1	
Others	14	50	50	

### Prognostic impact of PHD1 expression

**Fig. 3:**
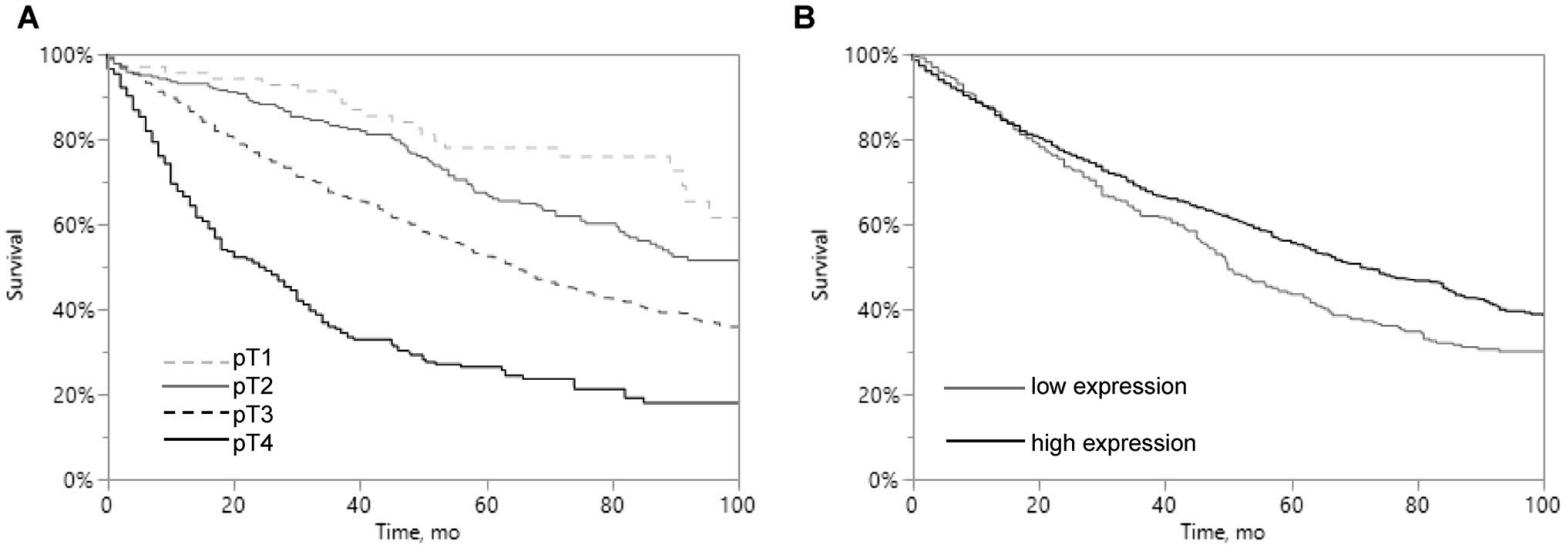
Kaplan–Meier analysis of PHD1 protein expression in primary CRCs. **A** Association between overall survival of patients and tumor stage (*p* < 0.0001). **B** Association between clinical outcome in CRCs and PHD1 expression (*p* < 0.0001)

As expected, high tumor stage was associated with poor patient survival (*p* < 0.0001; Fig. 3A). Interestingly, low PHD1 expression was significantly related to unfavorable outcome (*p* = 0.0011; Fig. 3B).

Multivariable analysis, including tumor stage, histological type, and PHD1 staining, revealed tumor stage and histological type (*p* < 0.0001 each), but also PHD1 staining (*p* = 0.0202) to be independent prognostic markers in our patient cohort (Table 3).

**Table 3:** Multivariate analysis including PHD1 immunostaining results

Included parameters	*p* value
Tumor stage	*p* < 0.0001
Histological type	*p* < 0.0001
PHD1 staining	*p* = 0.0202

### Association between PHD1 expression and Ki67 labeling index in CRCs

To analyze the correlation between PHD1 and Ki67 expression in CRCs, we used our previously published data on Ki67 expression in CRCs (Melling et al. 2016). We were able to show that strong PHD1 expression was significantly associated with high Ki67 expression levels in CRC (*p* < 0.0001).

## Discussion

Our results show that loss of PHD1 expression identifies a subset of CRC patients with poor overall survival and might be a promising prognostic marker in CRC patients. For this study, we were able to take advantage of our large TMA with 1800 CRC samples and corresponding pathological and survival data. To our knowledge, this is the largest CRC TMA reported on in the literature.

The data derived from this study demonstrate a decrease in PHD1 expression from normal colorectal epithelial cells to cancer cells. This observation is in accordance with earlier studies using IHC in 93 CRCs (Xie et al. 2012) as well as RT-PCR and western blotting in 90 CRCs (Rawluszko et al. 2013). In other studies, both decrease or increase of PHD1 levels have been described in malignant relative to corresponding benign tissue (Andersen et al. 2011) (Giatromanolaki et al. 2008) (Couvelard et al. 2008) (Gossage et al. 2010). For example, low PHD1 staining was found in lung cancers (Andersen et al. 2011) (Giatromanolaki et al. 2008) and high PHD1 staining in pancreatic endocrine tumors (Couvelard et al. 2008) and pancreatic adenocarcinomas (Gossage et al. 2010). These differences may be due to different interactions between PHD1 and tissue-specific carcinogenic pathways. This suggestion is supported by in vivo and in vitro data on PHD1 in various cancers describing either tumor-suppressive or oncogenic functions of PHD1 in dependence of the cell-specific signaling pathways. For example, loss of PHD1 expression in breast cancer as well as overexpression of PHD1 in lung cancer cells both lead to suppressed cell proliferation and tumor formation (Zhang et al. 2009) (Xie et al. 2014).

Our results revealed a high level of PHD1 expression in benign colorectal tissue and early stage cancers. The correlation between pT stage and immunohistochemistry is mainly driven by pT1 cancers showing strong PHD1 staining. Thus, the finding of decreased PHD1 expression in advanced pT stages argues for a role of PHD1 loss during colorectal tumorigenesis. This assumption is underlined by our result that decreased PHD1 staining was linked to cancers with advanced tumor stage and poor prognosis. The mechanism of how lack of PHD1 drives aggressive tumor phenotype and poor prognosis in CRCs remains elusive. However, our data fit well to earlier functional data on PHD-HIF signaling pathways in colorectal cells, suggesting that PHD1 might rather play a tumor-suppressive than an oncogenic role in CRCs. Functional studies revealed that colorectal carcinoma cell lines stably expressing PHD1 grew significantly slower and formed smaller tumors in nude mice as compared with control cells (Erez et al. 2003). Moreover, ectopic expression of PHD1 suppressed accumulation of HIF1α (Erez et al. 2003), which has been—when overexpressed—associated with mortality in colorectal cancer patients (Baba et al. 2010) (Novell et al. 2014). Very similarly to our results, PHD3, from the same family of proteins as PHD1, was found to be decreased in CRC cells and associated with higher tumor grade and metastasis (Xue et al. 2010). Knockdown of PHD3 in this study led to increased resistance of CRC cells to tumor necrosis factor alpha. The authors suggest a tumor-suppressive role of PHD3 in colorectal cancer which is corroborated by our results for PHD1. Therefore, if the mechanism in the PHD1–HIF pathway, responsible for the supposed tumor suppression via PHD1, were to be found, a novel target for individualized colon cancer therapy may become available for selected patients.

Furthermore, PHD1 has been suggested to play a role in resistance to chemotherapy in CRC (Deschoemaeker et al. 2015). Silencing of PHD1 has been shown to prevent p53 activation upon chemotherapy in different CRC cell lines, thereby inhibiting DNA repair and favoring cell death (Deschoemaeker et al. 2015). In accordance with this observation, knockdown of PHD1 has been demonstrated to sensitize CRC to 5-FU in mice (Deschoemaeker et al. 2015). Thus, these authors suggested that PHD1 might be part of the resistance machinery in CRC (Deschoemaeker et al. 2015). This, too, may eventually allow for PHD1 targeting as a promising specific therapeutic approach in certain patients.

In summary, we were able to show that reduced PHD1 expression is linked to a subset of CRCs with aggressive tumor features and is an independent predictor of poor prognosis in a very large patient cohort. Thus, our results provide more evidence for a prominent tumorigenic role of the PHD1–HIF signaling pathway in CRC cells. Further clarification of this pathway is needed to define a potential therapeutic role of PHD1 targeting in the future.

## Conclusion

Here, we demonstrate that PHD1 expression is decreased in malignant as compared to benign colorectal tissue. Moreover, low PHD1 expression is associated with worse clinical outcome in CRCs and may serve as an independent prognostic biomarker in CRCs and may potentially become a therapeutic marker in the future.

## Data Availability

The data that support the findings of this study are available from the corresponding author, NM.

## Abbreviations

TMA: Tissue microarray
CRC: Colorectal cancer
HIF: Hypoxia inducible factor
PHD: Prolyl hydroxylase
PHD1: Prolyl hydroxylase 1
IHC: Immunohistochemistry
RT-PCR: Reverse transcription polymerase chain reaction
