# Correction: Decreased expression of prolyl hydroxylase 1 is associated with poor prognosis in colorectal cancers

**DOI:** 10.1007/s00432-024-05729-y

**Published:** 2024-04-16

**Authors:** Nathaniel Melling, Julia Grass, Matthias Reeh, Michael Tachezy, Marco Blessmann, Jakob R. Izbicki, Katharina Grupp

**Affiliations:** 1 Department of General, Visceral and Thoracic Surgery, University Medical Center Hamburg-Eppendorf, Hamburg, Germany; 2 Department of Plastic, Reconstructive and Aesthetic Surgery, University Medical Center Hamburg-Eppendorf, Hamburg, Germany

**Correction: Journal of Cancer Research and Clinical Oncology (2023) 149:7579–7585** 10.1007/s00432-023-04717-y

In this article, the author group was incorrectly published as

Nathaniel Melling, Julia Grass, Matthias Reeh, Michael Tachezy, Marco Blessmann, Felix Nickel, Thilo Hackert, Katharina Grupp

The correct author group is listed below,

Nathaniel Melling, Julia Grass, Matthias Reeh, Michael Tachezy, Marco Blessmann, Jakob R. Izbicki, Katharina Grupp

The original article has been corrected.

